# Hand in the snow

**DOI:** 10.1186/1758-2946-3-S1-O1

**Published:** 2011-04-19

**Authors:** Colleen Fitzpatrick

**Affiliations:** 1 Huntington Beach, CA, USA

## 

As reported by MSNBC, CNN, and in newspapers worldwide:

On March 12, 1948, at 9:14 pm, Northwest Flight 4422 en route from Shanghai, China to La Guardia Airfield in New York, slammed into Mount Sanford, a 16,237-foot high peak located in a remote area of Alaska, 200 miles northeast of Anchorage.

The 30 military personnel onboard, including six crew members and 24 merchant mariner passengers, were killed instantly. Nothing was done to recover the wreck. Because of its remote location, the crash site was abandoned, the debris digested within a few days by the active glacier into which it fell. It would take fifty years, and the efforts of two commercial airline pilots to reach the scene of the accident. It would take another ten years and a team of world class DNA experts, forensic genealogists, and fingerprint experts to identify the frozen human forearm and hand that was found by the pilots, well-preserved by the glacier for half a century.

By September 2008, 28 of the 30 passengers had been eliminated by DNA analysis, fingerprint matching, or both. Only two more passengers remained on the list, the two most difficult to locate family members for who could serve as references for DNA analysis.

Our search for the family of passenger No 29 was featured in newspapers around the world, including USA Today and the Washington Post. Francis J Van Zandt was born in 1911 in Vermont, the son of a father from upstate New York and a mother who was an obscure Irish immigrant named Margaret Conway. This lecture describes how we achieved the impossible and found a DNA match for the arm and hand in the snow, finally laying to rest the 30 victims of Northwest Flight 4422.

